# When she is Standing Left, she Might be Blamed. Responsibility Attribution for Sexualized Violence Moderated by Rape Myth Acceptance and Benevolent Sexism

**DOI:** 10.1177/10778012221108420

**Published:** 2022-08-30

**Authors:** Katharina T. Halicki, Robin Hauser, Michaela Wänke

**Affiliations:** 1 26573 University of Mannheim, Mannheim, Germany

**Keywords:** victim blaming, rape myth acceptance, benevolent sexism, pragmatic relevance, positioning bias

## Abstract

The present research contributes to the literature on victim blaming in cases of sexualized violence. Our findings show that even subtle cues, such as positioning in a picture, can influence blame attribution, particularly for people who are motivated to do so. In our experimental study we could show that with increasing rape myth acceptance as well as with increasing benevolent sexism, participants assigned more responsibility for later occurring sexualized violence to a woman displayed on the left-hand side compared to a woman displayed on the right-hand side of a picture.

## Introduction

Crime victims often suffer twice. Not only are they harmed by the criminal act, but also they might be blamed for bearing at least some responsibility for what happened (Bohner, 2001; Maes, 1994; Van den Bos & Maas, 2009). This victim blaming is particularly likely in cases of sexualized violence (Bieneck & Krahé, 2011; Bohner, 2001; Bohner et al., 2009; Grubb & Turner, 2012; Süssenbach et al., 2017). Various factors, such as structural racism (Miller, 2019), victim gender, and sexual orientation (Wakelin & Long, 2003), or the relationship between perpetrator and victim (Viki et al., 2004) influence how people ascribe responsibility for sexual assaults to the perpetrator or the victim. Another factor contributing to victim blaming is how sexual assaults are verbally described, for example in the media. In this regard, verb voice has been shown to affect how responsibility is attributed. For example, rape can be described in active voice: “the man raped the woman” or in passive voice: “the woman was raped by the man” or even “the woman was raped.” Passive voice emphasizes the victim compared to the perpetrator. This focus on the victim obscures the agency and responsibility (Henley et al., 1995) and shifts causal attributions for the assault from the perpetrator to the victim (Bohner, 2001).

The present research looks at another facet of how subtle variations in the way information is presented affect responsibility attributions in sexualized violence. Namely, we look at the position of the female victim and the male perpetrator in a picture, presumably taken before the assault occurred. Various research from different domains suggests that whether a person is depicted on the left-hand side or on the right-hand side in a social interaction is neither arbitrary nor inconsequential (Chatterjee et al., 1995, 1999; Maass et al., 2009;Suitner et al., 2017). More concretely, the left versus right position in a picture will determine the focus of attention, salience, and causal inferences (Lassiter et al., 2002; Taylor & Fiske, 1975; Taylor et al., 1979). As this affects responsibility judgments (Rempala & Bernieri, 2005), we claim that, just like active versus passive voice, positioning affects who is blamed in the context of sexualized violence. Namely, we assume that the left position in a picture is more strongly associated with victim blaming than the right position. We will review these thoughts in the next paragraph. Following this, we will turn to possible moderators, namely rape myth acceptance and benevolent sexism.

### Positioning and Victim Blaming

There are several reasons to assume that the position in a picture may shift the responsibility for a sexual assault from the perpetrator to the victim: firstly, many studies attested prominence and relevance to the left position in a picture. Predominately when forming a visual image of a verbally described scene the grammatical subject is placed on the left-hand side of a picture (e.g., Halicki et al., 2021; Maass et al., 2014). The bias for the left is only found in cultures writing from left-to-right and reverses in cultures with a right-to-left script (Maass et al., 2014). This parallels the word order as in most languages the grammatical subject precedes the object (Dryer, 2005). Importantly, the grammatical subject “is the part on which we wish to focus, it is the center of the discourse” (Geminiani et al., 1995, p. 1566) and is used “when the ‘whom’ of a story is more important than the ‘who’” (Bremner, 1980, p. 8). This has the consequence that the person who is pragmatically most relevant for an interaction is expected to be depicted on the left-hand side of a picture (Halicki et al., 2021). Indeed, Halicki et al. (2021) showed that it is the pragmatic relevance of a protagonist in a sentence that determines the positioning.

One may speculate whether there is also the reverse relationship: not only are more important protagonists placed on the left side (in cultures with left-to-right script direction) but persons placed on the left are also more prominent and salient. This in turn may affect further attributions like responsibility judgments as people have a strong tendency to attribute causality and control to salient actors (Taylor & Fiske, 1975; Taylor et al., 1979) and people's causal attributions are influenced by what captures their visual attention (Lassiter et al., 2002). Indeed, Rempala and Bernieri (2005) see salience as a possible boost for biased responsibility judgments and therefore for victim blaming. Thus, the left position in a picture may put the spotlight on the depicted person and therefore we assume that it could affect victim blaming.

Secondly and somewhat related, according to the Spatial Agency Bias (SAB), the left position in a picture is associated with an agency (Suitner & Maass, 2016). In the SAB literature, agency is mostly referred to acting and having the capacity to act (Suitner & Maass, 2016). For example, when participants read or hear scene descriptions of a social interaction involving two protagonists (e.g., “exchanging a gift” or “Tom kicks Georg”), they tend to position the one performing the action (the agent) on the left-hand side of the recipient of the action (Chatterjee et al., 1995; Maass et al., 2014). The bias that the agent is predominantly positioned on the left-hand side is interpreted as support for the SAB and the correspondence between the agency and the left position (Suitner & Giacomantio, 2012; Suitner & Maass, 2016). Initially considered as the consequence of brain structures and hemisphere specialization, Maass and colleagues (Maass & Russo, 2003; Suitner & Maass, 2016) showed that this spatial bias originates from the direction of the script in one's culture. Again, the bias for the left is only prevalent in cultures writing from left to right reverses in cultures with right-to-left script (Maass & Russo, 2003; Maass et al., 2014, Suitner & Maass, 2016).

Further, grammatical functions also play a role: due to the frequent use of active voice, the thematic agent of a sentence is commonly presented in the function of the grammatical subject. In active voice sentences, the person functioning as the grammatical subject is also the acting person, the thematic agent. In combination with a left-to-right script, this leads to a frequent exposure of the thematic agent on the left in written texts and possibly causes an association between the left position and the agentic role (Maass et al., 2014; Suitner & Maass, 2016). Moreover, Maass et al. (2014, for an overview, see Suitner & Maass, 2016) also argue that because of the ubiquity of reading and writing their direction shapes the mental representation of other actions as well. If the script is evolving from left to right, activities are generally expected to follow a left to right trajectory and vice versa.

Clearly, the concept of agency is not restricted to merely performing an action. Agentic traits such as masculinity and power are also assigned to the left side and with a rightward orientation (Suitner et al., 2017). One may also conceptualize agency as the power and the ability to control or cause a situation (Bandura, 1989; Hitlin & Elder, 2007), agency also refers to the capacity of forethought and the capacity to imagine alternative possibilities (Bandura, 1989; Emirbayer & Mische, 1998). In the context of sexualized violence, Henley et al. (1995) understand responsibility attributions as a form of agency. As already mentioned, research on positioning based on relevance finds evidence that the person who is pragmatically most relevant for an interaction is expected to be depicted on the left-hand side of a picture and not necessarily the person who is performing the action (Halicki et al., 2021). This relevance account of the spatial bias could either be understood as a broader concept of agency or it could be an independent spatial bias that works additively.

Both accounts, salience/relevance and agency due to the left position in a picture, lead us to our first hypothesis.

Hypothesis 1: In cases of sexualized violence, participants will ascribe more responsibility to women who are displayed on the left-hand side of the perpetrator than to women who are displayed on the right-hand side of the perpetrator.

It should be noted that this is extending the present evidence on spatial positioning that mainly focused on which person is positioned on the left-hand side but not what is interpreted from positions in pictures (for exceptions see Maass et al., 2007 and Suitner et al., 2017; however, focusing on leftward vs. rightward orientation instead of positioning).

Of course, the willingness to blame the female victim in cases of sexual violence is likely to correlate with one's general attitude toward women. We assume that people who are generally inclined to blame the victim are particularly sensitive to use any cues that give them a reason to do so whereas people less inclined may not react to such subtleties as the position in a picture (in accordance with motivated reasoning, see Kunda, 1990). Attitudes that promote victim blaming are rape myth acceptance and benevolent sexism.

### Rape Myth Acceptance

Rape myths are based on beliefs about rape that aim at justifying sexual violence against women (Süssenbach et al., 2017). Bohner and colleagues defined rape myths as “descriptive or prescriptive beliefs about rape (i.e., about its causes, context, consequences, perpetrators, victims, and their interaction) that serve to deny, downplay or justify sexual violence that men commit against women” (Bohner, 1998, p. 14; Gerger et al., 2007, p. 423). Therefore, accepting rape myths is associated with trivializing sexualized violence against women and predicts stronger antivictim as well as pro-perpetrator judgments in rape scenarios (Bohner, 1998; Bohner et al., 2009: Süssenbach et al., 2017).

Furthermore, increasing rape myth acceptance leads to shifting attention to the victim and away from the perpetrator. For example, participants who highly agreed with rape myths tended more strongly to search for more information on the victim (Süssenbach et al., 2017). In the same vein when presented with pictorial material, participants with high rape myth acceptance tend to avoid looking at the perpetrator (Süssenbach et al., 2017). Further, they might also shift attention to the victim by using passive language when writing about rape cases (Bohner, 2001).

Assuming that people with high rape myth acceptance are motivated to search for and be sensitive to information that potentially confirms their views and justifies blaming the victim of a sexual assault we expect that these people are more likely to be influenced by the position in the pictures in assigning responsibility.

Hypothesis 2: Rape myth acceptance will moderate the relationship between the position of the women in the picture and responsibility attribution in cases of sexualized violence. The higher attribution of responsibility for women depicted on the left side of the perpetrator compared to the right side will be stronger for participants with high levels of rape myth acceptance.

### Benevolent Sexism

Another factor that might possibly influence positioning effects on responsibility judgments is benevolent sexism, which is a subtype of sexism that reflects stereotypes toward women in traditional roles (Glick & Fiske, 1996). In contrast to hostile sexism that reflects negative attitudes toward women, benevolent sexist attitudes are “sexist but subjectively positive and affectionate toward women” (Viki & Abrams, 2002, p. 289). It pertains to traditional gender roles according to which women are the fairer but weaker sex. Therefore, women are pure and need to be protected. For example, people with benevolent sexist beliefs would agree that women should be rescued before men when it comes to a catastrophe (Eckes & Six-Materna, 1999). However, these beliefs also imply the notion that women need to behave accordingly and in a way that allows men to protect them (Abrams et al., 2003). Therefore, benevolent sexists blame women for everything that is happening under circumstances in which traditional gender roles are violated (Abrams et al., 2003).

Gender stereotypes that are in line with benevolent sexist beliefs contradict agency attributions (defining agency by acting and having the capacity to act, as well as broader agency definitions, including the perception of power and control, self-efficacy beliefs, and the capacity of forethought, see Bandura, 1989; Emirbayer & Mische, 1998; Hitlin & Elder, 2007; Suitner & Maass, 2016). For example, relying on others for protection and accepting to be the weaker sex, implies an idea of passivity and low efficacy beliefs and could be seen as little agentic. Additionally, Suitner and colleagues argued that masculinity is an agentic trait (Suitner & Maass, 2008; Suitner et al., 2017), which is therefore also associated with the left side and a rightward trajectory (Suitner et al., 2017). In line with this idea, Suitner et al. (2017) find evidence that people high in benevolent sexism associate males more strongly with the left position and a leftward orientation. Accordingly, displaying women on the left-hand side and therefore with a rightward profile contradicts the typical orientation in which women are presented according to people clinging to stereotypical beliefs (Maass et al., 2009; Suitner et al., 2017).

Consequently, a woman depicted on the left-hand side violates gender-role expectancies for people high in benevolent sexism. Since it is expected that people who believe in benevolent sexist stereotypes blame women when they are not acting according to their stereotypical beliefs (Abrams et al., 2003), we assume that benevolent sexism moderates the positioning effect on victim blaming.

Hypothesis 3: Benevolent sexism will moderate the relationship between the position of the women in the picture and responsibility attribution in cases of sexualized violence. The higher attribution of responsibility for women depicted on the left side of the perpetrator compared to the right side will be stronger for participants with high levels of benevolent sexism.

## Method

### Participants

We limit this study to a male sample as prior research found victim blaming in the case of sexual violence more pronounced for men (see for example Henley et al., 1995; Rempala & Bernieri, 2005).

Participants were recruited via social networks (i.e., Facebook and Instagram) and students of the University of Mannheim, Germany, were also recruited through Sona Systems (a digital participation management for psychology studies at the University of Mannheim). One hundred and three German-speaking participants completed the online study. We excluded six participants that did not indicate being male or who were not native speakers. Further, we excluded three participants who were familiar with languages that have a right-to-left reading script, and 11 participants who did not categorize themselves as being heterosexual, since interpretations of the benevolent sexism measure could be misleading for nonheterosexual participants (e.g., “Men are imperfect without women”). Our final sample consists of 84 participants^1^ (*M*_age_ = 27.76, *SD*_age_ = 9.78). Students of the University of Mannheim could receive course credits for participating. No other compensation or reward was granted.

### Design and Procedure

The study was advertised with the title: “What happened afterwards?” Participants were informed that they would see pictures of a scene and that later an incident of sexualized violence occurred following the situation depicted. We had explicitly warned participants that the study would confront them with the topic of sexualized violence.

Each participant viewed eight scenes depicting a woman and a man (four scenes showing a woman on the left-hand side of the man and four scenes showing the woman on the right-hand side of the man). A mirrored version of each picture existed, so that in one version, the woman was on the left-hand side of the man, and in one version the woman was on the right-hand side of the man in the picture. Every participant saw only one version of each picture: in total, four pictures with the woman on the left-hand side and four pictures with the woman on the right-hand side, which of the pictures showed the woman on the left-hand side and which showed the woman on the right-hand side was counterbalanced between participants and pictures were shown in randomized order. Participants’ task was to rate responsibility for the later incident of sexualized violence. Subsequently, rape myth acceptance and benevolent sexism were recorded. Following demographic questions, the subjects were informed about the aim of the study. The Ethics Committee of the University of Mannheim had approved the study.

### Material

#### Picture Set

Great care was taken to ensure that the man and the woman were clearly depicted on the left-hand or right-hand side of the picture. No other persons were displayed in the background of the picture. The original eight pictures were retrieved from Adobe Stock.^2^ A mirrored duplicate was made from each picture, in order to create two versions of the pictures (one with the woman on the left-hand side and one with the woman on the right-hand side). Each picture set per participant consisted of four pictures with the women on the left-hand side and four pictures with the woman on the right-hand side. In total, each participant saw eight different scenes.

The atmosphere in the picture varied between being flirty, relaxed, or aggressive. The interactions depicted on the flirty and relaxed pictures varied between having an alcoholic drink (e.g., beer, wine, and cocktails) and having a hot drink. From the pictures it is not possible to clearly determine whether the people are in a relationship or not and in which places the scene takes place. Previous evidence shows that context effects (e.g., alcohol consumption and relationship status) affect victim blaming (Grubb & Turner, 2012; Viki et al., 2004). We have no hypothesis on how these contextual effects might interfere with positioning effects and our moderators. It could be possible that alcohol consumption prevents floor effects for victim blaming and that we only find the hypothesized effects for pictures with alcohol involved. On the other hand, it is possible that alcohol consumption overrides other influences on victim blaming, diminishing potential positioning effects. Therefore, we also used other relaxed or flirty scenes where the protagonists have a nonalcoholic drink, namely hot drinks such as coffee (based on the fact that there are served in a cup). We further chose three more aggressive scenes to broaden the scope of potential interactions in which sexualized violence occurs. One of the aggressive scenes could represent a verbal argument between a couple, another an argument in an office where the man comes quite close to the woman who leans toward a window and the third actually involves aggressive bodily contact where the man holds the woman with both hands.

#### Dependent Variable

Responsibility for the incident of sexualized violence was operationalized with four items that were rated on a seven-point rating scale ranging from “don't agree at all” to “fully agree.” The items were^3^: “The woman probably sent the man misleading signals”; “The woman has contributed through her behavior to the occurrence of sexualized violence”; “From the man's point of view, the subsequent incident was probably consensual”; and “The man bears the responsibility for the incident of sexualized violence.” The last item was inverted and mean values of all four items served as responsibility rating (*M* = 3.03, *SD* = 1.44, Cronbach's alpha = .80, see Table 1). We counterbalanced between participants whether the two items mentioning the woman or the two items mentioning the man were asked first.

**Table 1.** Descriptive Data and Bivariate Correlations.

Variable	*M*	*SD*	Cronbach's Alpha	Bivariate correlations		
				Position^1^	Responsibility ratings	Rape myth acceptance
Responsibility ratings^2^	3.03	1.44	.80	.04		
Rape myth acceptance	2.51	0.69	.80	−.01	.43*	
Benevolent sexism	2.94	0.82	.86	.01	.22*	.56*

*Note.* ^1^Coding: woman on the right-hand side = −1, woman on the left-hand side = 1; ^2^mean and standard deviations for all pictures: *indicates *p *< .001.

#### Rape Myth Acceptance

Rape myth acceptance was rated on the German *Vergewaltigungsmythenakzeptanz*-*Skala* (VMAS; Bohner, 1998). The 20 items were rated on a seven-point rating scale from “does not apply at all” to “absolutely applies.” An example item is “Women often challenge rape through their outward appearance or behavior.”^4^ (*M* = 2.51, *SD* = 0.69, Cronbach's alpha = .80, see Table 1).

*Benevolent sexism.* The German version (Eckes & Six-Materna, 1999) of the subscale from the Ambivalent Sexism Inventory (Glick & Fiske, 1996) served as a measure of benevolent sexism. The subscale contains 11 items that were rated on a five-point scale ranging from “does not apply at all” to “absolutely applies.” An example item is “Women should be cared for and protected by men”^5^ (*M* = 2.94, *SD* = 0.82, Cronbach's alpha = .86, see Table 1).

## Results

**Figure 1.**
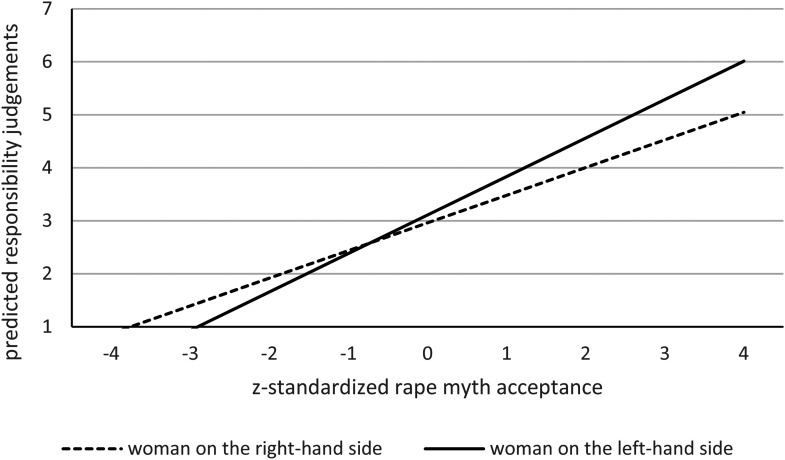
Predicted responsibility judgments for the model including rape myth acceptance as moderator.

**Figure 2.**
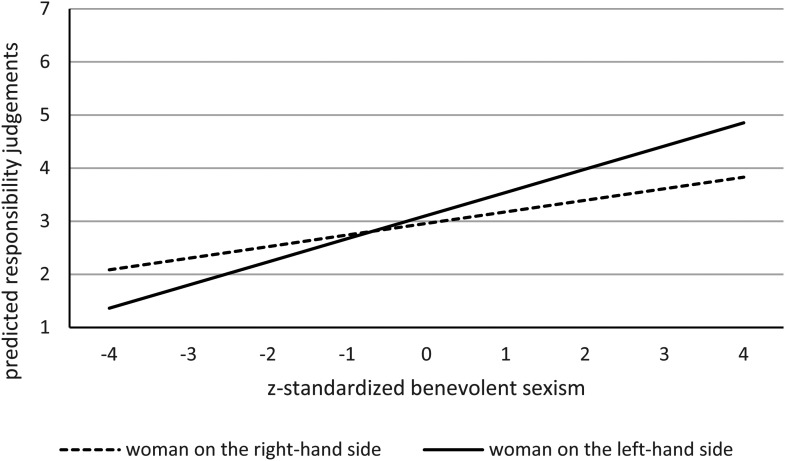
Predicted responsibility judgments with benevolent sexism as moderator.

Data analysis was separated for the two moderators rape myth acceptance (see Table 2 & Figure 1) and benevolent sexism (see Table 3 and Figure 2; a correlation between the two moderators; *r* = .59, *p *< .001, see Table 1).

**Table 2.** Fixed Effects Estimates of the Linear Mixed-Model with Rape Myth Acceptance as Moderator.

Fixed effect	B	SE	*df*	*t*	*p*	95% CI
Intercept	3.04	0.09	81.07	34.55	<.000	[2.86, 3.21]
Position^1^	0.07	0.04	587.51	1.72	.085	[−0.01, 0.16]
Rape myth acceptance^2^	0.62	0.09	81.07	7.09	<.000	[0.45, 0.80]
Position × rape myth acceptance^2^	0.10	0.04	590.20	2.34	.019	[0.02, 0.19]

*Note.* ^1^Coding: woman on the right-hand side = −1, woman on the left-hand side = 1. ^2^*z*-Standardized.

**Table 3.** Fixed Effects Estimates of the Linear Mixed-Model with Benevolent Sexism as Moderator.

Fixed effect	*b*	SE	*df*	*t*	*p*	95% CI
Intercept	3.02	0.11	81.04	28.74	0.000	[2.82, 3.24]
Position^1^	0.07	0.04	584.95	1.72	0.086	[−0.01, 0.16]
Benevolent sexism^2^	0.33	0.11	81.01	3.10	0.003	[0.12, 0.54]
Position × benevolent sexism^2^	0.11	0.04	587.08	2.51	0.012	[0.02, 0.19]

*Note.* ^1^Coding: woman on the right-hand side = −1, woman on the left-hand side = 1. ^2^ *z*-Standardized.

### Rape Myth Acceptance

We ran a linear mixed-model with mean responsibility ratings for each of the eight pictures as a dependent variable (see Table 2 and Figure 1). To account for interindividual differences, we included random intercepts per participant. An effect coded variable as predictor indicated which picture presented the woman on the left-hand side and which picture presented the woman on the right-hand side (position: woman on the right-hand side = −1, woman on the left-hand side = 1; included as a fixed effect). Further, we included rape myth acceptance (*z*-standardized) and the interaction term (position × *z*-standardized rape myth acceptance) as fixed effects.

As predicted in Hypothesis 1, participants tend to attribute more responsibility to a woman displayed on the left. However, this difference fell below conventional standards of significance (*b* = 0.07, *SE* = .04, *t*(587.51) = 1.72, *p* = .085, 95% CI [−0.01, 0.16]). Further, we find a significant effect for rape myth acceptance: with increasing rape myth acceptance, participants attributed significantly more responsibility to the women (*b* = 0.62, *SE* = .09, *t*(81.07) = 7.09, *p *< .001, 95% CI [0.45, 0.80]).

More importantly, in line with our Hypothesis 2, we find a significant interaction between position and rape myth acceptance (*b* = 0.10, *SE* = .04, *t*(590.20) = 2.34, *p* = .019, 95% CI [0.02, 0.19]). With increasing rape myth acceptance, the higher responsibility attribution for women on the left compared to the right position becomes more pronounced (see Table 2 and Figure 1).

### Benevolent Sexism

Again, we ran a linear mixed-model with mean responsibility ratings for each of the eight pictures as a dependent variable and included random intercepts per participant (see Table 3 and Figure 2). An effect coded variable as a predictor indicated which picture presented the woman on the left-hand side and which picture presented the woman on the right-hand side (position: woman on the right-hand side = −1, woman on the left-hand side = 1; included as a fixed effect). This time we included benevolent sexism (*z*-standardized) and the interaction term (position × *z*-standardized benevolent sexism) as fixed effects.

Again, the effect of positioning fell below conventional standards of significance (*b* = 0.07, *SE* = .04, *t*[584.95] = 1.72, *p* = .086, 95% CI [−0.01, 0.16]). We further find a significant effect of benevolent sexism. With increasing benevolent sexism scores, participants attribute more responsibility to the women (*b* = 0.33, *SE* = .11, *t*[81.01] = 3.10, *p* = .003, 95% CI [0.12, 0.54]).

In line with Hypothesis 3, a significant interaction between position and benevolent sexism emerged (*b* = 0.11, *SE* = .04, *t*[587.08] = 2.51, *p* = .012, 95% CI [0.02, 0.19]). With rising benevolent sexism, higher responsibility attribution for women on the left compared to the right position becomes more pronounced (see Table 3 and Figure 2).

### Exploratory Analysis

To cover a wide variety of incidences in which sexualized violence could happen, we had decided to vary the atmosphere of the scenes presented. However, research has shown that some of these aspects (e.g., alcohol consumption) also affect victim blaming (Grubb & Turner, 2012; Richardson & Campbell, 1982; Romero-Sánchez et al., 2018). Therefore, we categorized our pictures according to three categories (alcohol involved^6^; aggressive atmosphere^7^; and relaxed scenes with hot drinks^8^ (e.g., coffee, see Appendix Table A1). To compare the overall victim blaming tendency between the scene categories, we ran a linear mixed-model with mean responsibility ratings as a dependent variable and a variable coding the three scene types as a predictor. This analysis reveals a significant difference between the scene types (*F*[2, 578] = 185.78, *p *< .001). Pairwise comparisons with Bonferroni correction for multiple comparisons reveal that the aggressive scenes (*M*_aggressive_ = 2.19, *SE* = 0.12) result in lower victim blaming than the scenes with alcohol consumption (*M*_alcohol_ = 3.53, *SE* = 0.12) or where the protagonists have a hot drink (*M*_hot drink_ = 3.54, *SE* = 0.13). The scenes with alcohol consumption and hot drinks did not differ.^9^

Additionally, to test our hypotheses for the different scenes, we ran the linear mixed models described above (including positioning and our moderators) separately for each scene category. We decided to analyze all three groups separately, even though we did not find responsibility differences between alcohol and hot drink consumption, since it is discussed in the literature whether the type of drink has an effect on victim blaming (Richardson & Campbell, 1982). In none of the separate analyses a positioning effect was found. For scenes involving alcohol we do not find an effect for the interaction between position and rape myth acceptance (see Appendix Table A2) or position and benevolent sexism (Appendix Table A3) either. For aggressive scenes, however, the interaction between position and rape myth acceptance is marginally significant (Appendix Table A4) and the interaction between position and benevolent sexism remains significant (Appendix Table A5). In the remaining scenes (hot drink), again, the interaction between position and benevolent sexism remains significant (Appendix Table A6), but the interaction between position and rape myth acceptance does not reach significance (Appendix Table A7).

## Discussion

The aim of the current study was to test whether rather subtle cues such as the position in a picture could affect responsibility judgments for a later occurring incidence of sexualized violence and if explicit attitudes such as rape myth acceptance and benevolent sexism moderate this effect. Contrary to our hypothesis that participants attribute more responsibility to women depicted on the left-hand side of the perpetrator compared to being depicted on the right-hand side, the effect was rather small and fell below the conventional statistical significance level (Hypothesis 1). Nevertheless, in line with our two other hypotheses, we found evidence that rape myth acceptance (Hypothesis 2) and benevolent sexism (Hypothesis 3) moderate the effect of positioning on victim blaming. With increasing rape myth acceptance as well as with increasing benevolent sexism, participants assigned more responsibility for later occurring sexualized violence to a woman displayed on the left-hand side compared to a woman displayed on the right-hand side. Additionally, replicating previous research, increasing rape myth acceptance (Süssenbach et al., 2017), as well as increasing benevolent sexism (Abrams et al., 2003) are associated with higher blame attribution toward the victim of sexualized violence.

Our findings have implications for victim blaming research. Finding that positioning has an effect on responsibility judgments (moderated by rape myth acceptance as well as benevolent sexism) provides further evidence for the way how the presentation of a woman influences victim blaming. As explained, positioning might be a means to direct attention to the victim (similar to the effect of passive voice use, see Bohner, 2001; Henley et al., 1995). Therefore, in victim blaming research, care must be taken to see if the victim is emphasized as this might foster victim blaming. When using pictures, one idea could be to control for these effects by counterbalancing the position of the protagonists between or within participants.

Suitner et al. (2017) have shown that positioning might be a means to change gender stereotypes in a positive way. For example, by counter-stereotypical positioning, that is, displaying women frequently with a rightward-oriented profile, participants learned new associations which resulted in reduced benevolent sexism. However, in our experiment, positioning on the left-hand side had negative consequences for the displayed women, at least for participants with increasing benevolent sexism and rape myth acceptance. Hence, positioning may lead to different judgments, depending on context. For example, we could expect that if agentic behavior is positively associated, a woman could profit from a left position. However, when agentic behavior could be associated with negative aspects, the left position in a picture could have negative consequences. Further research could investigate under which conditions other consequences (e.g., influences on empathy or compassion) could be provoked by positioning or a general focus of attention.

Our pictures showed different types of scenes. We categorized them into aggressive scenes, scenes in which alcohol consumption was involved and scenes in a relaxed atmosphere where the protagonists have a hot drink. Research has shown that alcohol consumption enhances victim blaming (Grubb & Turner, 2012; Richardson & Campbell, 1982; Romero-Sánchez et al., 2018) and therefore it was reasonable to expect a difference between the scenes. Indeed, our exploratory analysis provides evidence for this idea. Compared to the aggressive scenes, alcohol consumption resulted in higher responsibility ratings of the women. However, we did not find differences between the scenes where the protagonists drink alcohol or a hot drink. Additionally, drinking a hot drink was also associated with higher responsibility ratings compared to aggressive scenes. The effect of lower victim blaming in the aggressive scenes was most pronounced in the one scene involving aggressive bodily contact with the man holding the woman with both hands. There the facial impression of the woman clearly signals rejection and leaves little room for interpretation of whether this could be a “misleading” signal. However, also the other less drastic scenes show a reduction. This finding adds to the victim blaming literature, providing evidence that the atmosphere in which the victim and perpetrator are depicted has an influence on the way sexualized violence is seen. It is possible that independent of victim blaming tendencies, a victim that looks disgusted and refusing any contact is less likely to be judged being responsible for sexualized violence. Further, although not central to the present research, it is noteworthy that even in these scenes rape myth acceptance and benevolent sexism increased victim blaming.

Looking at our hypotheses tests within the different scene types, position alone had no effect in either of the scenes. In aggressive scenes as well as in scenes involving a nonalcoholic hot drink, benevolent sexism had more impact on victim blaming when the woman was positioned on the left. A parallel but much weaker and nonsignificant pattern emerged for rape myth acceptance. For scenes involving alcoholic drinks, no such patterns were observed. How strong such rather subtle cues like positioning are and if they are able to override other cues is not clear yet. It is possible that positioning is only used as a cue for victim blaming when other cues are missing. Alcohol consumption might function as one of these cues (Grubb & Turner, 2012; Richardson & Campbell, 1982; Romero-Sánchez et al., 2018) or potentially overrides any effects that are more subtle.

### Practical Implications

Our findings have practical implications concerning the way in which positioning affects gender stereotypes and victim blaming tendencies. In times when “pictures speak louder than words” (Lee et al., 2015), several news agencies publish their news on pictorial-based social networks such as Instagram or Facebook. As a consequence of this, information is often published in combination with a picture which could either be a picture from the actual event or situation or if no picture is available, stock pictures are used. The way a woman who experienced sexual violence is depicted might influence the perception of the viewers (see, e.g., Schwark & Bohner, 2019; Schwark, 2017). When news agencies use stock pictures of an interaction between a woman and a man, to write about rape or about statistics of sexualized violence, the positioning within the picture might influence responsibility judgments and therefore how the role of the victim is seen by the viewers. For example, just using a picture with a woman on the left-hand side and a man on the right-hand side might evoke the thought for some people that women are responsible when they are the victim of sexualized violence.

Further, let us assume a victim of sexual violence had posted a picture of a happy scene with the latter perpetrator on a social network. The picture might be used as evidence in a trial. Besides the atmosphere of the depicted scene (Grubb & Turner, 2012), also positioning could possibly influence responsibility judgments and thus the degree of penalty. One idea to reduce positioning effects could be to show two versions of the picture: the original as well as a mirrored version.

The question of whether positioning or a general focus of attention enhances victim blaming is especially relevant since it is discussed whether news agencies and social media should refrain from focusing on the offender of violent acts and instead also name the victims and tell their stories. This has the purpose to commemorate the victims and avoid depersonalization (i.e., done with the hashtags #saytheirnames or #sayhername). This approach is reasonable as it refrains from offering a platform for offenders of violence, especially if the violence has a racist background. However, it could have unintended negative side effects. Knowing more details about the victim, for example, that the person was adventurous and was living a worry-free life, some people might interpret this information as if the person was reckless, naïve, or agentic and self-determined. As seen in our results, these interpretations might foster victim blaming. Thus, more information on the victim provides a platform for accusing the victim that if they would have behaved differently, they would have been able to prevent being a victim. Further research could test under which conditions or for which types of violence emphasizing the victim results in enhanced victim blaming.

### Limitations and Future Research

The results showed that there was a positioning effect for people high in rape myth acceptance and benevolent sexism. However, the predicted main effect of positioning was weak, at best only marginally significant, and disappeared when looking only at subsets of the scenes. Our exploratory analysis with the subsamples of different scene types gives preliminary evidence that positioning effects, as well as the interaction of positioning and rape myth acceptance or positioning and benevolent sexism, are not robust. However, we had no hypotheses on how the scene types could interfere with positioning and did not control for it or other potential influences of the depicted scenes. Information about the familiarity between victim and perpetrator (Viki et al., 2004), alcohol consumption (Grubb & Turner, 2012; Richardson & Campbell, 1982; Romero-Sánchez et al., 2018); or sexual orientation (Wakelin & Long, 2003) might override or alter the positioning effect. Therefore, the exploratory analysis has to be interpreted cautiously. A systematic analysis of context effects could provide more evidence. This information could be given either within the picture or by giving contextual information by using vignettes. Further, our results are limited to one study. Clearly, more evidence is needed.

In our paradigm, participants receive only sparse information about sexual assault. This was done in order to omit active and passive voice formulations (e.g., “the woman was raped by the man” or “the man raped the woman”) which could have additional effects (Henley et al., 1995). Therefore, participants only learn that following the situation depicted, an incident of sexualized violence occurred. Further research could therefore investigate the impact of additional information on the positioning effect. For example, other studies in the context of victim blaming often use a mock jury paradigm where participants receive information about the sexual assault in form of vignettes (Bohner, 2001; Temkin & Krahé, 2008). It could be tested whether active or passive voice scene descriptions reduce or amplify positioning effects.

Besides benevolent sexism, also hostile sexism might have an influence on perception and judgments of sexualized violence. We decided to investigate the impact of benevolent sexist beliefs, as the previous literature suggests that benevolent sexism but not hostile sexism affects victim blaming in acquaintance rape scenarios (Abrams et al., 2003). Nevertheless, since hostile sexism and benevolent sexism are positively correlated (Abrams et al., 2003) and hostile sexism affects rape proclivity, future research might investigate conditions under the two different types of sexism that affect positioning effects on victim blaming.

The present study employed a within-participant design to provide more statistical power. It cannot be excluded that the positioning variations in the within-design made participants more sensitive to the positioning. Therefore, different research designs (between-participant designs and within-participant designs) could be used in replication studies.

In line with previous research in the area of victim blaming, we restricted our research to a male participant sample. However, research exists that also women engage in victim blaming (Cowan, 2000; Ståhl et al., 2010). Therefore, further research could aim at testing whether positioning also predicts victim blaming for a female sample and if gender differences occur for this effect.

## Conclusion

This article contributes to the literature on victim blaming in cases of sexualized violence. Our findings show that even subtle cues, such as positioning in a picture, can influence the attribution of responsibility. Particularly for people who are motivated to do so. Therefore, our results contribute to the area of implicit stereotyping in combination with explicit attitudes such as benevolent sexism and rape myth acceptance. Just as using passive voice when describing sexual violence shifts attention from perpetrator to victim and thereby heightens responsibility attributions, the left position presumably marks relevance and guides attention toward the depicted person with the same result.

## Notes

1. Calculating appropriate power analyses and effect sizes for linear mixed models is not trivial (Brysbaert & Stevens, 2018). Therefore, we decided to use the more conservative test that does not account for repeated measures, namely an *F*-test for fixed effects and interaction, using G*power (Faul et al., 2007) which reveals a sample size of *N* = 90 (*f* = .3, α = .05, 1 − β = .8). The resulting sample size was somewhat smaller than the planned sample size. Note however that the planned sample size was based on conservative assumptions. As revealed by a sensitivity analysis, the actual sample size still allowed us to detect an effect of *f* = .31 with 1 − β = .8 and α = .05.

2. Picture numbers: 49940810, 105504728, 114641608, 122725006, 171041435, 173982997, 177899638, and 199353287.

3. Original items in German: “Die Frau hat dem Mann vermutlich missverständliche Signale gesendet.”; “Die Frau hat durch ihr Verhalten dazu beigetragen, dass es zum Vorfall sexualisierter Gewalt gekommen ist.”; “Aus Sicht des Mannes was der nachfolgende Vorfall vermutlich einvernehmlich.”; “Der Mann trägt die volle Verantwortung dafür, dass es zu dem Vorfall sexualisierter Gewalt gekommen ist.”

4. Original item in German: “Oft fordern Frauen eine Vergewaltigung durch ihre äußerliche Erscheinung oder ihr Verhalten heraus.”

5. Original item in German: “Frauen sollten von Männern umsorgt und beschützt werden.”

6. Picture numbers: 105504728, 122725006, and 199353287.

7. Picture numbers: 49940810, 114641608, and 171041435

8. Picture numbers: 173982997 and 177899638.

9. *M*_aggressive_ – *M*_alcohol_ = −1.34, *p *< .001; *M*_aggressive_ – *M*_hot drink_ = −1.34, *p *< .001; *M*_alcohol_ – *M*_hot drink_ = −0.01, *p* = 1.00.

## Appendix

See Tables A1 to A7.

**Table A1.** Means and Standard Deviations of the Individual Scenes.

Subset	Adobe stock number	Short description^1^	*M*	*SD*
Alcohol involved	199353287	Drinking a cocktail at a bar	3.36	1.28
	105504728	Drinking beer at a bar	3.76	1.39
	122725006	Drinking wine at the outdoor area of a restaurant	3.48	1.38
			3.53	1.35
Aggressive atmosphere	171041435	Argument between a couple	2.47	1.21
	49940810	Argument between coworkers	2.22	1.06
	114641608	Man harassing woman at the street	1.89	1.00
			2.19	1.12
Relaxed scenes with hot drinks	173982997	Having a hot drink in a coffee	3.35	1.49
	177899638	Having a hot drink at home or a coffee	3.73	1.35
			3.54	1.43

*Note.* ^1^Descriptions are just for first orientation, pictures leave more room for interpretation about the location or the relationship status.

**Table A2.** Subset Scenes with Alcohol Consumption. Fixed Effects Estimates of the Linear Mixed-Model with Rape Myth Acceptance as Moderator.

Fixed effect	*b*	SE	*df*	*t*	*p*	95% CI
Intercept	3.51	0.11	80.41	31.54	<.000	[3.29, 3.73]
Position^1^	0.04	0.05	188.52	0.77	.439	[−0.07, 0.15]
Rape myth acceptance^2^	0.74	0.12	80.41	6.14	<.000	[0.50, 0.98]
Position × rape myth acceptance^2^	−0.04	0.06	186.58	−0.60	.546	[−0.15, 0.08]

*Note.* ^1^Coding: woman on the right-hand side = −1, woman on the left-hand side = 1. ^2^*z*-Standardized.

**Table A3.** Subset Scenes with Alcohol Consumption. Fixed Effects Estimates of the Linear Mixed-Model with Benevolent Sexism as Moderator.

Fixed effect	*b*	SE	*df*	*t*	*p*	95% CI
Intercept	3.51	0.13	80.83	27.16	<.000	[3.25, 3.77]
Position^1^	0.04	0.06	181.84	0.80	.424	[−0.06, 0.15]
Benevolent sexism	0.34	0.13	80.84	2.64	.010	[0.08, 0.59]
Position × benevolent sexism	0.03	0.06	188.03	0.58	.560	[−0.08, 0.15]

*Note.* ^1^Coding: woman on the right-hand side = −1, woman on the left-hand side = 1. ^2^ z-Standardized.

**Table A4.** Subset Scenes with Aggressive Atmosphere. Fixed Effects Estimates of the Linear Mixed-Model with Rape Myth Acceptance as Moderator.

Fixed effect	*b*	SE	*df*	*t*	*p*	95% CI
Intercept	2.19	0.08	82.27	26.45	<.000	[2.03, 2.36]
Position^1^	0.06	0.06	209.76	1.05	.295	[−0.05, 0.17]
Rape myth acceptance^2^	0.56	0.09	83.11	6.24	<.000	[0.38, 0.74]
Position × rape myth acceptance^2^	0.12	0.06	202.37	1.88	.062	[−0.01, 0.24]

*Note.* ^1^Coding: woman on the right-hand side = −1, woman on the left-hand side = 1. ^2^*z*-Standardized.

**Table A5.** Subset Scenes with Aggressive Atmosphere. Fixed Effects Estimates of the Linear Mixed-Model with Benevolent Sexism as Moderator.

Fixed effect	*b*	SE	*df*	*t*	*p*	95% CI
Intercept	2.19	0.10	80.55	22.78	<.000	[2.00, 2.38]
Position^1^	0.06	0.06	198.06	0.96	.341	[−0.06, 0.17]
Benevolent sexism	0.29	0.09	80.09	3.10	.003	[0.11, 0.48]
Position × benevolent sexism	0.13	0.06	198.27	2.27	.024	[0.02, 0.24]

*Note.* ^1^Coding: woman on the right-hand side = −1, woman on the left-hand side = 1. ^2^*z*-Standardized.

**Table A6.** Subset Scenes with hot Drink. Fixed Effects Estimates of the Linear Mixed-Model with Benevolent Sexism as Moderator.

Fixed effect	*b*	SE	*df*	*t*	*p*	95% CI
Intercept	3.51	0.14	80.75	25.16	<.000	[3.24, 3.79]
Position^1^	0.03	0.09	128.09	0.40	.690	[−0.14, 0.20]
Benevolent sexism	0.35	0.14	80.76	2.53	.013	[0.07, 0.62]
Position × benevolent sexism	0.21	0.09	147.46	2.21	.029	[0.02, 0.39]

*Note.* ^1^Coding: woman on the right-hand side = −1, woman on the left-hand side = 1. ^2^*z*-Standardized.

**Table A7.** Subset Scenes with hot Drink. Fixed Effects Estimates of the Linear Mixed-Model with Rape Myth Acceptance as Moderator.

Fixed effect	*B*	SE	*df*	*t*	*p*	95% CI
Intercept	3.51	0.12	80.63	28.54	<.000	[3.27, 3.76]
Position^1^	0.02	0.08	138.12	0.28	.779	[−0.14, 0.19]
Rape myth acceptance^2^	0.75	0.13	80.79	5.64	<.000	[0.49, 1.01]
Position × rape myth acceptance^2^	0.13	0.09	145.62	1.33	.185	[−0.06, 0.31]

*Note.* ^1^Coding: woman on the right-hand side = −1, woman on the left-hand side = 1. ^2^*z*-standardized.
